# The Effect of a Pain Management Empowerment Program on Nursing Care Quality in Pediatric ICUs: A Quasi‐Experimental Study

**DOI:** 10.1155/prm/2605293

**Published:** 2026-06-28

**Authors:** Zhila Tahmasb Zamanian, FatemehSadat SeyedNematollah Roshan, Narges Rahmani

**Affiliations:** ^1^ Department of Nursing, TeMS. C., Islamic Azad University, Tehran, Iran, azad.ac.ir; ^2^ Department of Nursing and Midwifery, Bab.C., Islamic Azad University, Babol, Iran, azad.ac.ir

**Keywords:** empowerment, nurse, pain management, pediatric special care department, quality of care

## Abstract

**Objective:**

High‐quality patient care relies on nurses’ adherence to professional nursing standards. Effective pain management reduces disease severity, accelerates recovery, decreases the length of hospital stay, and improves care quality indicators. This study aimed to examine the effect of empowering nurses in pain management on the quality of care in pediatric intensive care units.

**Methods:**

This quasi‐experimental study was conducted in two pediatric intensive care units in Tehran, Iran. Using a two‐stage cluster randomization method, 60 nurses were allocated to test and control groups (30 per group). The intervention consisted of a structured pain management training program, including two 2‐h theoretical sessions and one practical bedside session covering pediatric pain assessment, standardized pain scales, and evidence‐based pain management strategies. Quality of nursing care was assessed at baseline and 1.5 months after the intervention using the QUALPAC questionnaire. Data were analyzed using SPSS Version 27 (*p* < 0.05).

**Results:**

At baseline, no significant differences were observed between the test and control groups in quality of care across physical, communication, and psychosocial dimensions (*p* > 0.05). After the intervention, the test group showed significant improvements in all dimensions: physical (86.73 ± 9.51 to 91.13 ± 8.62, *d* = 1.06), communication (43.43 ± 6.63 to 47.13 ± 4.89, *d* = 0.921), psychosocial (108.20 ± 13.30 to 123.70 ± 10.34, *d* = 1.56), and total quality of care (238.36 ± 27.00 to 261.26 ± 22.11, *d* = 1.53) (*p* < 0.001). No significant changes occurred in the control group (*p* = 0.06 − 0.584). Postintervention analyses revealed significant between‐group differences in all care‐quality dimensions and in total quality of care (*p* ≤ 0.05), with a higher proportion of participants reaching the completely favorable level in the test group.

**Conclusions:**

Nurse empowerment in pain management significantly improved the quality of care in pediatric intensive care units. These findings highlight the importance of structured educational interventions to enhance nursing performance in pain management.

**Summary**
• What does this paper contribute to the wider global clinical community?
◦ This study provides evidence that structured empowerment programs in pain management can significantly improve the quality of care delivered by nurses in pediatric intensive care units.
◦ The findings emphasize the importance of continuous education and skill‐building in enhancing nurses’ clinical performance and patient outcomes.
◦ The results may guide global healthcare systems in designing cost‐effective, scalable training interventions to strengthen pain management practices in pediatric critical care.

## 1. Introduction

Quality of care refers to the extent to which health services improve patient outcomes and ensure safe, effective practice [1]. In pediatric intensive care units (PICUs), where patients are highly vulnerable and require complex monitoring and interventions, the quality of nursing care directly influences safety, recovery, and overall clinical outcomes [2, 3].

Among the most persistent and under‐addressed challenges in PICUs is inadequate pediatric pain management [4]. Despite established clinical guidelines, evidence indicates that nurses’ knowledge, attitudes, and performance in pain assessment and management remain suboptimal, resulting in a significant gap between recommended standards and routine practice [5, 6]. Inadequate pain control in hospitalized children increases physiological and behavioral stress responses, may prolong mechanical ventilation, delay recovery, and extend hospital stay [7]. Furthermore, poorly managed pain can have long‐term developmental and psychological consequences [8]. In Iran, this gap appears particularly pronounced in PICUs, where nurses report limited continuing education opportunities, insufficient organizational support, and inconsistent implementation of standardized pain assessment tools and protocols [8, 9]. These findings suggest that the problem extends beyond knowledge deficits alone and reflects insufficient professional empowerment, including limited decision‐making support, reduced clinical autonomy, and weak institutional reinforcement of evidence‐based practice [10, 11].

Recent international evidence highlights that nurses’ empowerment, emotional intelligence, and professional quality of life are critical determinants of care quality in intensive and critical care settings. Supportive work environments and psychological empowerment have been significantly associated with higher perceived quality of nursing care and improved patient satisfaction in intensive care units [12–14]. In particular, Ayed reported that higher levels of emotional intelligence among neonatal intensive care unit nurses were positively associated with improved clinical decision‐making, suggesting that nurses’ cognitive and emotional capacities play a pivotal role in delivering high‐quality, patient‐centered care [14].

Despite this growing body of international evidence, limited empirical research has examined whether structured empowerment programs can specifically enhance nurses’ performance in pediatric pain management within Iranian PICUs. Accordingly, this quasi‐experimental study aims to evaluate the effect of a structured empowerment program on nurses’ pain management performance in PICUs in Iran. We hypothesize that nurses who participate in the Pain Management Empowerment Program will demonstrate significantly higher quality of nursing care in pediatric ICUs compared with nurses in the control group.

## 2. Methods

### 2.1. Design

This study used a quasi‐experimental, two‐group design with cluster allocation (hospital‐level assignment) to examine the effect of a structured pain management empowerment program on the quality of nursing care in PICUs. The study was conducted in two hospitals in Tehran, Iran, from 30th January 2024 to 1st September 2024.

Hospitals were allocated to the test and control group; however, because only two clusters were included, the study does not meet the methodological criteria for a fully powered cluster‐randomized controlled trial.

The use of only two clusters limits statistical power at the cluster level and restricts the generalizability of the findings beyond the participating hospitals. Therefore, results should be interpreted with caution, particularly regarding external validity.

### 2.2. Ethics Approval and Consent to Participate

Approval was obtained from the Research Council and Ethics Committee of the Medical Sciences Branch, Islamic Azad University, Tehran, Iran (IR.IAU.TMU.REC.1403.099, dated 20/02/2024). Although the study involved observational assessment during routine care without additional interventions or costs, written informed consent was obtained from parents or legal guardians of the children, and verbal informed consent was obtained from the nurses for tool validation. Ethical procedures ensured confidentiality, anonymity, and the right to withdraw.

### 2.3. Setting, Sampling, and Randomization

The research sample consisted of nurses working in PICUs. The sample size was calculated using G ∗ Power software, considering a confidence level of 95%, a type I error probability of 0.05, a test power of 80%, and an effect size of 0.5, resulting in 27 participants per group. Considering a 10% potential attrition rate, 30 nurses were recruited per group.

Randomization was conducted exclusively at the hospital (cluster) level rather than at the individual nurse level.

In the first stage, two hospitals were randomly selected from a list of 12 pediatric hospitals in Tehran. In the second stage, one hospital was allocated to the test group and the other to the control group using a dice roll (even = intervention and odd = control). Therefore, all nurses within each hospital received the same group assignment.

Within each hospital, 30 eligible nurses were selected through simple random sampling. To minimize contamination, hospitals were geographically separated, and participants were instructed not to share intervention materials with colleagues in the other group.

Because allocation occurred at the hospital level and only two clusters were included, this design may be susceptible to cluster‐level confounding and limit statistical independence between participants within the same hospital. Consequently, the findings should be interpreted with caution regarding internal and external validity.

Inclusion criteria:

− Bachelor’s degree or higher in nursing
− At least 3 months of experience in PICUs
− Willingness to participate in the study
− No participation in training courses related to pain management in the past year
− Written consent to participate

Exclusion criteria:

− Leaving work or relocating
− Illness or leave during the study period
− Failure to attend more than one training session
− Distortion or incomplete completion of questionnaires

### 2.4. Data Collection Tools

In this study, the data collection tools included three parts: a demographic information form, the Nurses’ Performance Checklist in Pain Management, and the QUALPAC Quality‐of‐Care Questionnaire.

#### 2.4.1. Nurses’ Performance Checklist in Pain Management

The Nurses’ Performance Checklist in relation to patient pain management consists of four questions that are completed by nurses in a self‐reported manner. The scoring of the tool is based on yes with a score of 2, somewhat with a score of 1, and no with a score. The range of scores varies from 0 to 8, with a score above the median, i.e., 4, indicating better performance and a score below the median indicating poor performance of nurses in the field of pain management.

The validity of this tool has been confirmed by Aflatonian and Rafati in Iran [15]. Mohammad Aliha et al. [16] reported the reliability of this tool using Cronbach’s alpha of 0.95 (16). In the present study, the validity of the questionnaire was assessed in practice using an expert panel (two faculty members and four clinical nurses) to determine whether the study topic had been adequately addressed in the questionnaire. In addition, to determine the reliability of the questionnaire, 10 percent of the sample size was selected and asked to complete the questionnaires, and the internal consistency of the questionnaire was calculated using Cronbach’s alpha coefficient, 0.740.

#### 2.4.2. QUALPAC Quality‐of‐Care Questionnaire

The Quality Patient Care Scale (QUALPAC) has been used in different versions across studies, with variations in the number of items due to cultural adaptation and research objectives. While the original instrument developed by Nabili and Bastani consisted of 65 items, modified versions with 68 or 72 items have also been applied [17]. In the present study, the 72‐item version was used, comprising three dimensions: physical care (26 items), communication (13 items), and psychosocial care (33 items). Items are rated on a five‐point Likert scale (never = 1, rarely = 2, sometimes = 3, most of the time = 4, and always = 5), and scores in each dimension are classified into three levels—unfavorable, relatively favorable, and favorable—according to the range of possible scores [18] (Table A1). The physical dimension ranges from 26 to 130, communication from 13 to 65, psychosocial from 33 to 165, and the total score from 72 to 360. This classification converts raw scores into ordinal levels, providing a clear framework for interpreting nursing care quality and evaluating the effectiveness of interventions. The QUALPACS assesses both technical and interpersonal aspects of care: Physical care reflects clinical nursing interventions, communication captures nurse–patient interactions, and psychosocial care addresses emotional support and respect for patients’ psychological needs, while the overall score reflects combined performance across these domains [19]. This tool has been used to evaluate the quality of nursing care since 1975 in America, England, and Nigeria [20]. In Iran, it is one of the most widely used tools to check the quality of nursing care, and so far, its validity has been confirmed in various studies. In the study of Bastani et al. [21], the reliability of the quality of care questionnaire was measured and reported with Cronbach’s alpha test of 0.91 (21).

In the present study, experts in pediatric nursing, pain management, and quality of care reviewed the items to ensure content relevance within the PICU context as part of the content validity assessment. For measuring reliability, Cronbach’s alpha was calculated for the entire tool as well as for each of its dimensions (physical, communication, and psychosocial). Total Cronbach’s *α* was 0.87 in this study. According to psychometric standards, a Cronbach’s alpha value greater than 0.70 is considered acceptable reliability [22].

In the present study, the QUALPAC questionnaire was self‐administered and completed independently and anonymously by the participating nurses to minimize response bias. Consequently, the findings from this tool may still be influenced by social desirability or overestimation of performance. In contrast, nurses’ performance in pain management was directly observed using a structured checklist, providing an objective assessment of actual practice. This distinction should be considered when interpreting the results.

### 2.5. Intervention

The intervention was carried out in 3 stages in the following order:

#### 2.5.1. Preintervention Stage

After obtaining permission from the ethics committee, the researcher went to two randomly selected hospitals. Then, 30 nurses in the test group hospital and 30 nurses in the control group hospital who met the criteria for entering the research were invited to attend an explanation meeting on a specific day and time in the hospital’s training hall. In the briefing session in both hospitals, the researcher introduced himself to the nurses. The purpose of the research was explained in simple language, ensuring the safety of the intervention, the confidentiality of information, and the right to withdraw from the research at any time. Written consent was obtained from them. In the preintervention phase, the questionnaires were administered to all participants. The researcher coordinated with the nurses in advance to provide the questionnaires on a predetermined day and time in a calm environment. All participants completed the questionnaires fully, with no missing or invalid responses. According to the review and analysis of the pretest, the researcher obtained the necessary information about the quality of care.

#### 2.5.2. Intervention Stage

The intervention consisted of a structured neonatal pain management program totaling 4 h, integrating theoretical instruction and supervised clinical practice.

#### 2.5.3. Theoretical Component (3 h)

The session began with introductions and orientation, where the researcher explained the structure of the program, including:

− Methods of lecture delivery, group discussions, and question‐and‐answer sessions
− Roles of session staff (session director, secretary, and blackboard secretary)
− Planning of practical session timings in consultation with participants

Next, nurses discussed their current practices in pain assessment and management in PICU settings. The researcher guided the discussion using the following questions:

− Have you ever examined and recorded the presence or absence of pain in a hospitalized child?
− How do you examine and record pain?
− Do you use a standardized tool to assess pain severity?
− Do you inform the physician if the child is in pain?
− What methods of pain relief do you use?
− Do you reassess pain after intervention?

After this discussion, the researcher introduced the COMFORT Pain Assessment Scale, explaining its six core indicators: alertness, calmness/agitation, respiratory response, physical movement, muscle tone, and facial tension (Table A2) [8]. For context, other commonly used neonatal pain scales, such as the NIPS, were briefly discussed, but COMFORT was emphasized as the primary operational tool.

Nurses received a brochure and instructions for scoring and documentation to review before the practical session.

The session also addressed common barriers to effective pain management, including workload constraints, inconsistent documentation, and uncertainty in analgesic administration. Evidence‐based strategies were emphasized, covering:

− Nonpharmacological strategies: oral sucrose administration (with timing and dosage considerations), swaddling, facilitated tucking, therapeutic positioning, non‐nutritive sucking, environmental modification (noise and light reduction), and parental presence when feasible
− Pharmacological principles: appropriate timing of analgesics, safe dosing, monitoring for adverse effects, and reassessment after administration

#### 2.5.4. Practical Component (1 h–Bedside Role‐Play)

During bedside practice, nurses applied the COMFORT scale in small groups (approximately three nurses per subgroup) using structured case scenarios and direct clinical practice with intubated infants.

Training focused sequentially on:

1. Systematic observation of COMFORT scale variables
2. Calculation and recording of total scores
3. Interpretation of score ranges (no pain, mild, moderate, and severe)
4. Selection of appropriate intervention strategies based on predefined thresholds

Each nurse practiced assessment and intervention on at least two intubated infants under direct supervision and immediate feedback from the instructor to ensure correct application.

During the bedside practical session, nurses performed hands‐on pain management activities under supervision. To ensure reproducibility and standardized assessment, a structured performance checklist was used (Table A3). This checklist guided nurses to:

1. Assess and record the presence or absence of patient pain.
2. Assess and record the type of pain.
3. Evaluate and record pain relief methods if pain is present.
4. Use standardized tools to assess pain intensity and document the scores accurately.

#### 2.5.5. Nurses’ Performance Was Observed, and Checklist Compliance Was Monitored

All sessions were delivered by a researcher with a Master’s degree in pediatric nursing and clinical experience in neonatal intensive care. A structured training outline and implementation protocol were developed before the intervention; a summarized version is presented in Table A4.

#### 2.5.6. Postintervention Stage

**FIGURE 1:**
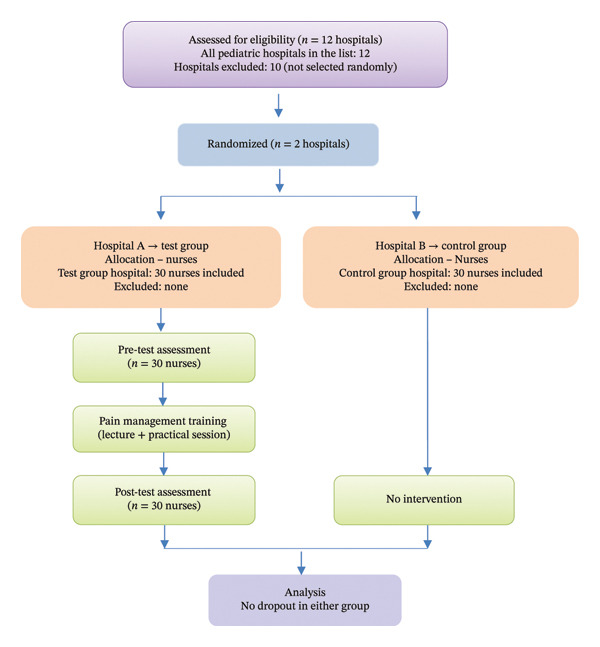
CONSORT flow diagram of nurse participation in the pain management training study.

After 1.5 months from the completion of the intervention in the test group and at the same time in the control group, the researcher provided the questionnaires to the participants as a post‐test after re‐coordinating with the nurses on the specified day and time. All questionnaires were fully completed, with no attrition. To reduce bias, data analysts were blinded, although nurses could not be blinded due to the nature of the intervention. The diagram of nurses’ participation in the pain management training study is presented in Figure 1.

### 2.6. Outcome Measures

The primary outcome of this study was nurses’ performance in pain management, evaluated via direct observation using the Nurses’ Performance Checklist. Secondary outcomes included overall nursing care quality, assessed through the self‐reported QUALPAC questionnaire across physical, communication, and psychosocial domains.

### 2.7. Data Analysis

Data were entered and analyzed using SPSS Version 27. Descriptive statistics (mean, standard deviation, frequency, and percentage) were calculated to summarize demographic characteristics. Baseline homogeneity between groups was assessed using chi‐square tests for categorical variables and independent *t*‐tests for continuous variables.

Normality was assessed using the Kolmogorov–Smirnov test. For normally distributed variables, parametric tests were used; otherwise, Mann–Whitney U and Wilcoxon signed‐rank tests were applied.

To evaluate the intervention effect, independent *t*‐tests were used to compare postintervention mean scores between groups. Paired *t*‐tests were applied to assess within‐group pre–post changes. For non‐normally distributed variables, Mann–Whitney U and Wilcoxon tests were used as appropriate.

Given the quasi‐experimental cluster allocation design and the inclusion of only two hospitals, analysis of covariance (ANCOVA) was not performed. We acknowledge that ANCOVA could provide additional adjustment for baseline scores; however, independent comparisons were used due to the limited number of clusters.

Effect sizes were calculated using Cohen’s *d* (0.2 = small, 0.5 = medium, 0.8 = large) [23]. Statistical significance was set at *p* < 0.05.

## 3. Results

Most nurses in the intervention and control groups were aged 30–39 and 40–49 years, respectively. Fisher’s exact test showed no significant differences in demographic variables between groups. The mean age was 38.83 ± 7.76 years in the intervention group and 36.60 ± 6.99 years in the control group, with no significant difference (*p* = 0.246). Additional demographic characteristics are presented in Table 1.

**TABLE 1:** Demographic characteristics of nurses in the test and control groups.

Variable		Test frequency (%)	Control frequency (%)	Statistical test	^∗^ *p* value
Age	20–29	5 (16.7)	7 (23.3)	*F* ^∗∗^	0.892
	30–39	13 (43.3)	9 (30.0)		
	40–49	9 (30.0)	12 (40.0)		
	50–59	3 (10.0)	2 (6.7)		

M ± Sd^∗∗∗^		38.83 ± 7.76	36.60 ± 6.99	s‐t‐test^∗∗∗∗^	0.246

Gender	Male	3 (10.0)	5 (16.7)	*F*	0.706
	Female	27 (90.0)	25 (83.3)		

Marital status	Single	18 (60.0)	20 (66.7)	χ^2^ ^∗∗∗∗∗^	0.789
	Married	12 (40.0)	10 (33.3)		

Education level	Bachelor	27 (90.0)	25 (83.3)	*F*	0.424
	Master	2 (6.7)	5 (16.7)		
	Ph.D	1 (3.3)	0 (0.0)		

Career position	Nurse	18 (60.0)	21 (70.0)	*F*	0.717
	Head‐nurse	9 (30.0)	7 (23.3)		
	Supervisor	3 (10.0)	2 (6.7)		

Work shift	Morning	7 (23.3)	3 (10.0)	*F*	0.303
	Evening	0 (0.0)	0 (0.0)		
	Night	2 (6.7)	2 (6.7)		
	Morning and evening	2 (6.7)	4 (13.3)		
	Evening and night	4 (13.3)	4 (13.3)		
	Circulating	17 (56.7)	15 (50.0)		

Clinical experience (year)	< 1	2 (6.7)	0 (0.0)	*F*	0.359
	2–5	5 (16.7)	8 (26.7)		
	6–10	6 (20.0)	5 (16.7)		
	11–15	8 (26.7)	12 (40.0)		
	> 16	9 (30.0)	5 (16.7)		

Pediatric intensive care unit experience	< 1	4 (13.3)	2 (6.7)	*F*	0.825
	2–5	7 (23.3)	9 (30.0)		
	6–10	10 (33.3)	8 (26.7)		
	11–15	7 (23.3)	7 (23.3)		
	> 16	2 (6.7)	4 (13.3)		
	Average	7 (23.3)	15 (50.0)		
	High	5 (16.7)	3 (10.0)		

^∗^Statistical significance.

^∗∗^Fisher’s exact test.

^∗∗∗^Mean ± standard deviation.

^∗∗∗∗^Independent samples test.

^∗∗∗∗∗^Chi‐squared.

The mean score of nurses’ performance in pain management in the intervention group was 1.84 ± 17.5 before the intervention and 1.35 ± 33.6 after the intervention, with a paired *t*‐test showing a significant improvement (*p* < 0.001). In the control group, the mean scores were 1.81 ± 5.03 at the first assessment and 1.07 ± 5.57 at the second assessment, with no significant difference between the two time points (*p* = 0.073).

Before the intervention, comparison of mean performance scores between the intervention and control groups using an independent *t*‐test showed no significant difference (*p* = 0.778), but after the intervention, the difference was statistically significant (*p* = 0.018).

Considering that the preintervention scores in the intervention group and the first assessment in the control group were above the median [4], nurses’ baseline performance in pain management can be considered good [16]. After the intervention, the mean score in the intervention group approached 8, corresponding to the highest performance criterion. Moreover, the effect size of the intervention on nurses’ performance, calculated using Cohen’s *d*, was greater than 0.4 [23], indicating a large effect (Table 2).

**TABLE 2:** The average performance score of pain management before and after the study in the two groups.

Group	Time		*p* value^∗∗^	ES^∗∗∗^	95% CI lower^∗∗∗∗^	95% CI upper^∗∗∗∗^
	Pre (mean ± SD)	Post (mean ± SD)				
Test	5.17 ± 1.84	6.33 ± 1.35	*p* < 0.001	0.81	0.39	1/22
Control	5.03 ± 1.81	5.07 ± 1.57	*p* = 0.073	0.34	−0.31	0/705
*p* value^∗^	*p* = 0.778	*p* = 0.018				

^∗^Independent *t*‐test (between groups).

^∗∗^Paired *t*‐test (within group).

^∗∗∗^Effect size by Cohen’s d.

^∗∗∗∗^Lower and upper confidence interval.

Before the intervention, there were no significant differences between the intervention and control groups in any dimension of the QUALPAC scale, including physical (*p* = 0.878), communication (*p* = 0.441), and psychosocial (*p* = 0.579) dimensions.

After the intervention, mean scores in the intervention group were higher than in the control group across all QUALPAC dimensions: physical (91.13 ± 8.62 vs. 86.26 ± 7.45, *p* = 0.023, Cohen’s *d* = 1.06), communication (47.13 ± 4.89 vs. 42.63 ± 4.31, *p* < 0.001, *d* = 0.921), psychosocial (123.70 ± 10.34 vs. 107.36 ± 8.49, *p* < 0.001, *d* = 1.56), and total quality of care (261.26 ± 22.11 vs. 236.26 ± 17.88, *p* < 0.001, *d* = 1.53).

Within‐group analysis using paired *t*‐tests showed significant pre–post increases in the intervention group in physical (86.73 ± 9.51 to 91.13 ± 8.62, *p* < 0.001), communication (43.43 ± 6.63 to 47.13 ± 4.89, *p* < 0.001), psychosocial (108.20 ± 13.30 to 123.70 ± 10.34, *p* < 0.001), and total quality of care (238.36 ± 27.00 to 261.26 ± 22.11, *p* < 0.001). No significant pre–post changes were observed in the control group (physical: *p* = 0.06; communication: *p* = 0.228; psychosocial: *p* = 0.102; total: *p* = 0.584) (Table 3).

**TABLE 3:** The average quality of care before and after the intervention in the two groups.

Dimensions	Time	Test mean ± SD	Control mean ± SD	*p* value^∗^
Physical	Before	86.73 ± 9.51	87.06 ± 7.08	0.878
	After	91.13 ± 8.62	86.26 ± 7.45	0.023
	ES^∗∗∗^	1.06	−0.358	

*p* value^∗∗^		< 0.001	0.06	

Communication	Before	43.43 ± 6.63	42.26 ± 4.89	0.441
	After	47.13 ± 4.89	42.63 ± 4.31	< 0.001
	ES^∗∗∗^	0.921	−0.353	

*p* value^∗∗^		< 0.001	0.228	

Psychosocial	Before	108.20 ± 13.30	106.56 ± 8.92	0.579
	After	123.70 ± 10.34	107.36 ± 8.49	< 0.001
	ES^∗∗∗^	1.56	0.309	

*p* value		< 0.001	0.102	

Total	Before	238.36 ± 27.00	235.90 ± 18.94	0.684
	After	261.26 ± 22.11	236.26 ± 17.88	< 0.001
	ES^∗∗∗^	1.53	0.101	

*p* value		< 0.001	0.584	

^∗^Independent t‐test.

^∗∗^Paired t‐test.

^∗∗∗^Effect size by Cohen’s d.

The distribution of quality‐of‐care levels across the QUALPAC dimensions before and after the intervention is presented in Table 4.

**TABLE 4:** Comparison of the level of quality of care before and after the intervention in the two groups.

Dimension	Time	Level of care	Test *n* (%)	Control *n* (%)	Between‐group *p* ^∗^
Physical	Before	Undesirable	2 (6.7)	0 (0)	0.099
		Relatively favorable	24 (80.0)	22 (73.3)	
		Completely favorable	4 (13.3)	8 (26.7)	
	After	Undesirable	0 (0)	0 (0)	0.038
		Relatively favorable	19 (63.3)	26 (86.7)	
		Completely favorable	11 (36.7)	4 (13.3)	

Within‐group *p* ^∗∗^		*p* value	0.083	0.157	

Communication	Before	Undesirable	1 (3.3)	0 (0)	0.708
		Relatively favorable	23 (76.7)	25 (83.3)	
		Completely favorable	6 (20.0)	5 (16.7)	
	After	Undesirable	0 (0)	0 (0)	0.047
		Relatively favorable	18 (60.0)	26 (86.7)	
		Completely favorable	12 (40.0)	4 (13.3)	

Within‐group *p* ^∗∗^		*p* value	0.008	0.317	

Psychosocial	Before	Undesirable	0 (0)	4 (13.3)	0.881
		Relatively favorable	25 (83.3)	24 (80.0)	
		Completely favorable	5 (16.7)	2 (6.7)	
	After	Undesirable	0 (0)	0 (0)	< 0.001
		Relatively favorable	14 (46.7)	29 (96.7)	
		Completely favorable	16 (53.3)	1 (3.3)	

Within‐group *p* ^∗∗^		*p* value	< 0.001	0.180	

Total quality of care	Before	Undesirable	0 (0)	0 (0)	0.209
		Relatively favorable	25 (83.3)	28 (93.3)	
		Completely favorable	5 (16.7)	2 (6.7)	
	After	Undesirable	0 (0)	2 (6.7)	< 0.001
		Relatively favorable	15 (50.0)	25 (83.3)	
		Completely favorable	15 (50.0)	3 (10.0)	

Within‐group *p* ^∗∗^		*p* value	0.002	0.564	

^∗^Mann–Whitney *U*.

^∗∗^Wilcoxon.

In the physical dimension, there was no significant between‐group difference before the intervention (*p* = 0.099). After the intervention, a significant between‐group difference was observed (*p* = 0.038). Within‐group changes were not statistically significant (intervention: *p* = 0.083; control: *p* = 0.157).

In the communication dimension, no significant difference was observed between groups before the intervention (*p* = 0.708). After the intervention, a significant between‐group difference was found (*p* = 0.047). Within‐group change was significant in the intervention group (*p* = 0.008) but not in the control group.

In the psychosocial dimension, no significant between‐group difference was observed before the intervention (*p* = 0.881). After the intervention, the between‐group difference was significant (*p* < 0.001). Within‐group change was significant in the intervention group (*p* < 0.001) but not in the control group (*p* = 0.180).

For total quality of care, there was no significant between‐group difference before the intervention (*p* = 0.209). After the intervention, the between‐group difference was significant (*p* < 0.001). Within‐group improvement was significant in the intervention group (*p* = 0.002) but not in the control group (*p* = 0.564).

## 4. Discussion

The findings indicate that the educational intervention substantially improved nurses’ performance in pain management. At baseline, both the intervention and control groups demonstrated acceptable competence, with scores above the median, suggesting generally adequate pre‐existing skills. Following the intervention, nurses in the intervention group approached the highest performance criterion. The improvement in nurses’ pain management performance in the current study aligns with emerging evidence indicating the ongoing challenges and the potential for educational interventions to strengthen clinical practice in pediatric critical care. A recent cross‐sectional study in PICUs reported that pain management remains complex, with a wide range of barriers and facilitators influencing nurses’ practices, highlighting the need for targeted training to enhance assessment and intervention behaviors [24]. Moreover, observational research on pediatric pain management found that while nurses often use nonpharmacological techniques, their application may be inconsistent, underscoring the importance of structured education to improve the regular use and confidence in effective pain interventions [25]. A large multicenter study of PICU nurses revealed that, despite relatively high use of pain scales for some patient groups, nurses’ satisfaction and confidence in pain assessment and management were low, and lack of knowledge was frequently identified as a barrier [7]. These contemporary findings support the conclusion that well‐designed educational programs, like the empowerment intervention in the present study, can play an important role in improving both performance and confidence in clinical pain management. Such interventions address knowledge gaps and practical barriers that, as systematic reviews suggest, are persistent obstacles to optimal pediatric pain care globally [26].

In the present study, empowerment interventions exhibited differential impacts across nursing care dimensions. While communication and psychosocial care demonstrated notable improvements, physical care showed limited change, likely due to its highly structured and protocol‐driven nature in PICUs. In these units, tasks such as monitoring vital signs, maintaining airway safety, and adhering to strict procedural guidelines are essential for patient safety but involve standardized procedures with minimal flexibility for individualized nursing decisions [21, 27]. As a result, short‐term educational interventions may increase awareness but are insufficient to produce substantial changes in deeply entrenched practices.

Contingency theory provides a framework to interpret these findings. The theory posits that intervention effectiveness depends on alignment with existing organizational structures. In highly regulated environments like PICUs, where care is guided by strict protocols, empowerment interventions may struggle to overcome ingrained practices without organizational support [28, 29]. System‐level support, repeated interventions, and reinforcement from leadership and staffing structures are crucial to achieving meaningful change in structured tasks. Hirao et al. similarly emphasize that technical nursing skills require continuous system‐level support to translate training into practice [27].

In contrast, communication and psychosocial dimensions rely more on interpersonal skills and professional judgment, allowing greater flexibility and responsiveness to empowerment interventions. Nurses in these areas can make independent decisions and tailor care based on patient and family needs [30, 31]. Our study aligns with this perspective, demonstrating substantial improvements in psychosocial care, including enhanced emotional support and facilitation of family participation, consistent with Mohebali et al. and Mcharo et al. [32, 33].

Overall, empowerment interventions significantly improved total quality of care, particularly in interpersonal and psychosocial domains, consistent with previous studies [34, 35]. These findings suggest that care areas less constrained by protocols are more amenable to short‐term educational and empowerment interventions.

### 4.1. Limitation

Several methodological and contextual factors limit the interpretation and generalizability of these results. First, the study used a cluster‐randomized design with only two hospitals (one per group), which represents a major methodological limitation. This design reduces the robustness of randomization, compromises the assumption of independence among participants within clusters, and limits the generalizability of the findings beyond the included hospitals. Consequently, the results should be interpreted with caution, particularly regarding internal validity and applicability to other PICU settings. Organizational differences, including staffing, workload, clinical routines, and supervisory support, may have independently influenced outcomes. Second, reliance on self‐reported measures introduces the risk of social desirability bias, potentially inflating perceived improvements, particularly in communication and psychosocial domains. Third, the short follow‐up period (1.5 months) constrains the detection of sustained or structural changes in protocol‐driven physical care. Additionally, unmeasured factors such as nurses’ intrinsic motivation, prior interest in psychosocial care, personality traits, and professional attitudes may have acted as confounders. Finally, informal communication between groups cannot be fully excluded, potentially reducing observed between‐group differences.

In conclusion, empowerment interventions are most effective in dimensions allowing autonomy and interpersonal engagement, while structured technical tasks require long‐term, repeated interventions and organizational reinforcement to achieve lasting improvements. Future research should evaluate repeated interventions and system‐level support to strengthen change in protocol‐driven care.

## 5. Conclusions

Empowerment interventions in PICUs can improve communication, psychosocial support, and overall nursing care quality, although the effect on physical care tasks appears limited due to their structured and protocol‐driven nature. These results suggest that targeted educational programs may enhance interpersonal skills, autonomy, and confidence among nurses in similar PICU settings. For meaningful improvement in the physical dimension, interventions may need to be longer, repeated, and supported by organizational structures that consider operational constraints and staffing. Future studies should employ observational assessments and longer follow‐up periods and account for institutional factors to better evaluate the impact of empowerment strategies.

## Author Contributions

Zhila Tahmasb Zamanian performed the data collection and drafted the manuscript. FatemehSadat SeyedNematollah Roshan supervised the analysis and research process. Narges Rahmani was responsible for the study concept and final approval of the submitted version.

## Funding

No funding was received for this manuscript.

## Disclosure

This article is a part of the pediatric nursing master’s thesis, which was approved with the number IR.IAU.TMU.REC.1403.099 dated 20/02/2024.

## Ethics Statement

Ethical approval was obtained from the Medical Sciences Branch, Islamic Azad University, Tehran, Iran (IR.IAU.TMU.REC.1403.099 dated 20/02/2024).

## Consent

All participants provided informed consent to participate in the study.

## Conflicts of Interest

The authors declare no conflicts of interest.

## Data Availability

The datasets generated and/or analyzed during the current study are not publicly available because individual privacy could be compromised. However, they are available from the corresponding author upon reasonable request.

## Appendix A

.

**TABLE A1:** Categorizing the quality of nursing care in QUALPACs tool.

Dimensions	Items	Favorable	Relatively favorable	Unfavorable
Physical	1–26	96–130	61–95.9	26–60.9
Communication	27–39	49–65	31–48.9	13–30.9
Psychosocial	40–72	123–165	78–122.9	33–77.9
Total	1–72	266–30	169–265.9	72–168.9

**TABLE A2:** COMFORT behavior scale and scoring form.

Date	Time	Observer
Alertness	• Deeply asleep (eyes closed, no response to changes in the environment)	❑ 1
	• Lightly asleep (eyes mostly closed, occasional responses)	❑ 2
	• Drowsy (child closes his or her eyes frequently, less responsive to the environment)	❑ 3
	• Awake and alert (child responsive to the environment)	❑ 4
	• Awake and hyperalert (exaggerated responses to environmental stimuli)	❑ 5

Calmness–Agitation	• Calm (child appears serene and tranquil)	❑ 1
	• Slightly anxious (child shows slight anxiety)	❑ 2
	• Anxious (child appears agitated but remains in control)	❑ 3
	• Very anxious (child appears very agitated, just able to control)	❑ 4
	• Panicky (child appears severely distressed, with loss of control)	❑ 5

Respiratory response (Score only in mechanically ventilated children)	• No spontaneous respiration	❑ 1
	• Spontaneous and ventilator respiration	❑ 2
	• Restlessness or resistance to the ventilator	❑ 3
	• Active breathing against the ventilator or regular coughing	❑ 4
	• Fighting against the ventilator	❑ 5

Crying (Score only in children breathing spontaneously)	• Quiet breathing, no crying sounds	❑ 1
	• Occasional sobbing or moaning	❑ 2
	• Whining (monotone)	❑ 3
	• Crying	❑ 4
	• Screaming or shrieking	❑ 5

Physical movement	• No movement	❑ 1
	• Occasional (3 or fewer) slight movements	❑ 2
	• Frequent (more than 3) slight movements	❑ 3
	• Vigorous movements limited to extremities	❑ 4
	• Vigorous movements, including torso and head	❑ 5

Muscle tone	•Muscles completely relaxed, no muscle tone	❑ 1
	•Reduced muscle tone, less resistance than normal	❑ 2
	•Normal muscle tone	❑ 3
	•Increased muscle tone and flexion of fingers and toes	❑ 4
	• Extreme muscle rigidity and flexion of fingers and toes	❑ 5

Facial tension	• Facial muscles completely relaxed	❑ 1
	• Normal facial tone	❑ 2
	• Tension evident in some facial muscles (not sustained)	❑ 3
	• Tension evident throughout facial muscles (sustained)	❑ 4
	• Facial muscles contorted and grimacing	❑ 5

*Note:* https://www.researchgate.net/publication/21611050_Assessing_Distress_in_Pediatric_Intensive_Care_Environments_The_COMFORT_Scale.

**TABLE A3:** Nurse performance checklist for pain management.

Item	Observation statement	Score		
		0	1	2
1	The nurse checks and records whether the patient has pain.			
2	The nurse identifies and documents the type of pain.			
3	If pain is present, the nurse evaluates and records the pain relief methods applied.			
4	The nurse uses a standardized tool to assess pain intensity and records the score correctly.			

*Note:* Instructions for Evaluator: (1) Observe the nurse performing each task at the bedside. (2) Assign a score based on whether the action is not performed (0), partially performed [1], or fully performed [2].

**TABLE A4:** Summary of study phases, intervention content, and assessment.

Phase	Activity	Duration	Learning objectives	Educational materials and methods	Instructor qualifications	Assessment tool
Preintervention	Briefing, consent, pretest	30–45 min	Explain the study purpose, ensure informed consent, clarify confidentiality, and withdrawal rights	Demographics + QUALPACs questionnaire	N/A	QUALPAC’s baseline score
Intervention (theoretical)	Lecture, group discussion, Q&A	3 h	1. Understand pediatric pain assessment principles 2. Apply standardized pain measurement tools (FLACC, Faces, Numeric Rating Scale) 3. Identify barriers to effective pain management 4. Apply evidence‐based pharmacological and nonpharmacological interventions 5. Document and report pain assessment	Slides, printed checklists, case scenarios, video demonstrations, interactive discussion, and role‐play	Master’s degree in Pediatric Nursing with practical PICU experience	Observation of participation and engagement
Intervention (practical)	Bedside role‐play	1 h	1. Perform an accurate pain assessment at the bedside 2. Implement age‐appropriate nonpharmacological interventions 3. Administer pharmacological interventions safely 4. Record pain scores correctly 5. Respond appropriately to changes in patient pain status	Standardized checklist, bedside demonstration, hands‐on practice on ≥ 2 patients, immediate feedback	Master’s degree in Pediatric Nursing with practical PICU experience	Observation of skills, accuracy, and documentation
Postintervention	Post‐test assessment	30–45 min	Evaluate the effect of the intervention on the quality of care	QUALPACs questionnaire	N/A	QUALPAC’s post‐test score, effect size (Cohen’s d)
